# Asking follow-up probes for selected responses in survey scales can affect responses to these items

**DOI:** 10.1371/journal.pone.0359515

**Published:** 2026-10-01

**Authors:** Daniel B. Wright

**Affiliations:** Department of Educational Psychology, Leadership, and Higher Education University of Nevada, Las Vegas, Nevada, United States of America; University of Health Sciences (Istanbul, Türkiye), TÜRKIYE

## Abstract

Having follow-up free response probes after forced choice survey questions allows researchers to assess the validity of the original choices. This can also be useful when psychological scales are administered as screening tools. The follow-up probes can be used to help decide if further diagnostic tests should be administered. A binary version of the General Health Questionnaire (GHQ-12) was given to an online sample (*n* = 1,048). Half answered the twelve GHQ questions in a binary format without follow-up probes. Half were asked with follow-up probes if they provided the psychologically concerning response. Those in the probe condition reported fewer psychologically concerning responses than those in the control condition. The difference between the probe and control groups increased as the survey went on. Researchers should be cautious comparing results from surveys that use selective follow-up probes with results from surveys that do not.

## 1. Background

Psychological scales are the foundation of much social and medical scientific research and are also used by practitioners when evaluating clients in these fields. A typical scale might ask a series of questions where the participant has to choose a single response alternative to summarize their belief. Asking a follow probe could help to check that the participant is interpreting the item as intended and as such help to evaluate the validity of the responses [1]. The follow-up probe can allow the participant to describe their belief in more detail than afforded with a closed forced choice response. Practitioners often use scales, particularly short forms of scales, with their clients as a screening device to help them decide if further diagnostic tests should be done [2]. Consider a practitioner asking the following item from the General Health Questionnaire (GHQ-12) [for a meta-analysis of this form, see 3]: “Felt capable of making decisions about things?”, and that they were offered these two response options: “More than usual” and “Less than usual.” Asking a person who responds “less than usual” to provide more information, like to describe an example, could help the practitioner decide how concerned they should be.

Follow-up probes are routinely used when constructing scales to make sure participants are interpreting the items correctly [4]. If used with an existing scale a key question, and the one asked in this study, is whether this affects participants’ responses to these questions. To address this question, it is useful to consider the cognitive aspects of survey responding [5–7]. Humans do not always seek optimal solutions to their tasks, but instead provide satisfactory solutions [8]. This *satisficing* has been observed in different types of surveys [9].

Recent innovations in survey methods may increase the likelihood that satisificing, as opposed to optimizing, occurs. An increasingly common method to recruit participants is to use companies like MTurk (www.mturk.com/) and Prolific (www.prolific.com/) to sample people from their lists of those willing to do tasks for small financial incentives. This is often referred to as online crowd sourcing. Messages are sent to thousands of people and the first people to respond are included in the sample. Researchers can specify filters on those who are initially approached. Some are basic demographic filters (e.g., here just people in the US are sampled), but others are based on the person’s performance on previous surveys, for example their percentage of satisfactory ratings on previous surveys. This can lead to a kind of professional participant as these tasks are always available. This could mean that some of those responding may answer in a manner so that their task is shorter than if they responded in a different way, increasing their hourly pay rate, but in a manner that allows them to get a satisfactory rating because researchers can limit who sees their studies to those with high proportions of satisfactory ratings. The prediction is that when follow-up probes are conditional on how a person answers and that when the probes are offered is predictable, people more often will respond so that they do not receive the follow-up probes.

The General Health Questionnaire (GHQ) [10] is the scale used in this study. This was chosen because it is a popular scale used by both researchers and practitioners across the health, medical, and social sciences. The GHQ comes in several formats, with different numbers of items and different response alternative sets. Here the GHQ-12 (12 items) with binary response format is used. The items describe a psychological experience like “Felt capable of making decisions about things?” and participants say whether they are experiencing this more or less than usual. Here, if the person says less than usual, this may suggest a psychological concern. A recent meta-analysis of several studies using the GHQ-12 shows good reliability [3], though questions have been raised about combining items that require reverse scoring with other items [11], an issue that has also been raised for other scales [12,13].

The prediction is that those who are offered a probe after each response where they provide the psychologically concerning response will be less likely to provide the psychologically concerning response thereby reducing the number of questions that they are asked and allowing them to finish more quickly (these are stated in the pre-registration). There are two primary predictions:

1 There will be a main effect with more people in the control condition giving the psychologically concerning response to the binary GHQ items.
2 The difference in reporting the psychologically concerning response, between the control condition and the probe condition, will increase as people are completing the survey. This would be measured by an interaction between the item order and the condition variable.

Predicting participants’ binary responses will be done with a binary multilevel logistic regression [14,15], with the responses nested within both participants and items, using the **lme4** package [16] within R [17]. Random effects will be included for the intercept for both participants and items. A main effect for the item order, from 1–12, will be added to the model. The main effect of condition will be added to evaluate the first prediction. Next, the interaction between item order and condition will be added to evaluate the second prediction. Details of this are included in a pre-registration, published at: https://osf.io/5uf72/overview.

## 2. Methods

### 2.1. Consent form

Prolific sent a note to potential respondents asking them if they wished to respond to a brief health questionnaire. The estimated time and payment they would receive was listed. If they choose to take part they were directed to the Qualtrics page where the study was located.

The first page of the Qualtrics form described the study in detail for potential respondents. It had sections on: purpose of the study, procedures (including randomizing people into conditions for the probes), benefits for participating, risks of participating, cost/compensation, confidentiality, and that it was voluntary participation. A toll-free number and an email address were provided for the UNLV Office of Research Integrity. There was no deception in this description. They were they asked to tick yes or no if they consented.

### 2.2. Power analysis

A power analysis was conducted to assess how likely it would be to find a significant (*p<*5%) effect in the predicted direction for prediction #2. The minimum effect the study is designed to detect is a 1% increase for each item in the difference between the control and the probe conditions for giving the psychologically concerning response (the probe condition proportion decreasing). The rbinom function in R was used to create the binary responses. The control group probability of responding with the psychologically concerning response stays constant. The probe group begins with the same expectancy, but decreases 1% with each trial. These data were simulated 2,000 times and the proportion of significant effects in the predicted direction was recorded. The code for this is provided in the pre-registration.

A sample size of *n* = 886 produced an estimated power of 95.45% (95% Wilson confidence interval of 94.45% to 96.28%). This interaction was chosen rather than the main effect as this effect is likely to be smaller. Because of exclusion criteria, the goal is to recruit 15% more so a minimum of 1,019. If someone starts the task they were allowed to continue and their data were included. This means the sample is larger than 1,019, and it depended on manually stopping the survey. The total sample taking part in this study was *n* = 1,059.

Everyone in the sample consented to take part. The exclusion criteria were being from a duplicate IP address, failing an in-built Qualtrics captcha question, and completing binary GHQ questions in an average of less than two seconds. The first criterion was in place because the second person at an IP address may have spoken with the previous person (6 were excluded for this reason). None failed the captcha question. Five responded, on average, faster than two seconds. This leaves a sample of *n* = 1,048.

Three demographics questions, related to gender, age, and race, were asked and any of these could be left blank. For gender, 50.42% responded female, 46.84% responded male, 2.27% responded non-binary, 0.09% responded other, and 0.38% preferred not to say or left it blank. The percentages providing each of the following age ranges (in years old) are in parentheses: 18 *−* 29 (27.01%), 30 *−* 39 (36.36%), 40 *−* 49 (18.41%), 50 *−* 59 (12.46%), 60 *−* 69 (3.97%), 70 *−* 79 (1.23%), and 80 or older (0.09%), with 0.38% preferring not to say or leaving the item blank. The sample is skewed towards younger people. For the race question, participants were presented with several options and told they were allowed to tick as many options as they felt appropriate, so the percentages sum to over 100%. These options and the percentages in parentheses are: White (75.07%), Black or African American (15.30%), Asian (9.73%), Native Indian or Native Alaskan (1.98%), Native Hawaiian Islander or Pacific Islander (0.09%), Middle Eastern (0.57%), and Other (2.93%), with less than 1% leaving it blank or ticking prefer not to say.

### 2.3. Design and materials

Participants were recruited by Prolific being told the study was a brief health questionnaire. The introduction described the study and that half of them would be randomly allocated to the probe condition where they would be asked follow-up probes depending on how they answered each of twelve two-alternative forced choice questions. After the exclusions described above, there were 536 (51.15%) in the control condition and 512 (48.85%) in the probe condition.

The twelve GHQ items are:

1. Been able to concentrate on what you’re doing?
2. Lost much sleep over worry?
3. Felt you were playing a useful part in things?
4. Felt capable of making decisions about things?
5. Felt constantly under strain?
6. Felt you couldn’t overcome your difficulties?
7. Been able to enjoy your normal day-to-day activities?
8. Been able to face up to your problems?
9. Been feeling unhappy and depressed?
10. Been losing confidence in yourself?
11. Been thinking of yourself as a worthless person?
12. Been feeling reasonably happy, all things considered?

Response times were record from when the item was on the screen to when the response was submitted. Two response alternatives for each of the form “more than usual” and “less than usual” were presented for each item on a single screen with the lower alternative one being the psychologically concerning response. The order of the items was kept constant to lessen random variation between the two conditions.

Those in the probe condition, if they provided the more psychologically concerning response, were prompted for example (for the first item) “You responded that you have been able to concentrate less than usual. Can you describe an example that you have experienced?” A free response box appeared and they were required to type at least 50 characters. The other probes were worded similarly, asking for an example.

Participants were thanked, debriefed, and returned to Prolific to receive $2 payment from Prolific. The median time was 212.50 seconds (about three and a half minutes), which equates with $33.88 per hour, though this does not account for the time on Prolific.

Ethical approval was obtained from the Office of Research Integrity – Research Protections (ORI-RP) at UNLV. They ruled that this project (UNLV-2026–110) exempt on April 21, 2026. The preregistration is at: https://osf.io/5uf72/overview. The survey is available at: https://unlv.co1.qualtrics.com/jfe/form/SV_dnhhX1HALYpRnJI.

## 3. Results

The data were downloaded from Qualtrics. The IP address was used in the exclusions. Variables that could be used for identification (e.g., IP address, location variables) were removed.

### 3.1. Predicting reports of psychological concern

Table 1 shows the proportion replying with the psychologically concerning response in the two conditions. The proportions for each of the trial orders, 1–12, are higher for the control condition than those offered a probe when giving the psychologically concerning response, as consistent with prediction #1. Given the sample size the width the 95% confidence intervals are approximately .08. The Wilson interval was used as recommended in Agresti [19]. Summing the number of psychologically concerning responses for all individuals, the mean for the control condition was 5.17 (*sd* = 4.23) and the mean for the probe condition was 3.26 (*sd* = 3.22). This difference is approximately *d* = 0.51 standard deviations.

**Table 1:** Proportions and 95% (Wilson) confidence intervals by the trial order and condition.

Trial	Control				Probe	
	est.	lb	ub	est.	lb	ub
1	.382	.342	.424	.381	.340	.424
2	.451	.410	.494	.381	.340	.424
3	.453	.412	.496	.326	.287	.368
4	.278	.242	.317	.158	.129	.192
5	.606	.564	.647	.467	.424	.510
6	.476	.434	.518	.289	.251	.330
7	.509	.467	.551	.314	.276	.356
8	.364	.324	.405	.125	.099	.156
9	.466	.425	.509	.285	.248	.326
10	.435	.393	.477	.166	.136	.201
11	.319	.281	.360	.131	.104	.163
12	.427	.386	.469	.234	.200	.273

The main inferential method for evaluating the two main predictions is a multilevel logistic regression [14,15] predicting whether the concerning response was given, allowing the intercept to vary by both the item and the person. The item order as a numeric variable was added, and then the experimental condition added. This evaluates the first prediction. Table 2 shows this improves the prediction (i.e., AIC and BIC lower than the previous run, *p*-value associated with the difference in models near zero). The 95% confidence interval for this effect was (−1.40, −0.82). The point estimate was *β* = −1.11.

**Table 2:** Statistics comparing different models predicting the probability of giving the psychologically concerning response.

Model	AIC	BIC	*Δχ* ^2^	df	*p*-value
intercept	11,906	11,928			
+item order	11,906	11,936	2.05	1	.153
+experimental factor	11,854	11,891	53.85	1	<.001
+interaction	11,766	11,811	89.81	1	<.001

The interaction between item order and the experimental condition was added to the model to evaluate the second prediction, that the difference between the probe and the control conditions increased during the duration of the scale. Table 2 shows this was observed (i.e., AIC and BIC lower the previous model, *p*-value associated with the difference in models near zero). The 95% bootstrap confidence interval [20,21] for this effect was (−0.17, −0.11). The point estimate was *β* = −0.14. Figure 1 shows both the predicted effects for the probability of giving the psychologically concerning response. As the scale continues, the probability of those giving the psychologically concerning approaches zero.

**Fig 1:**
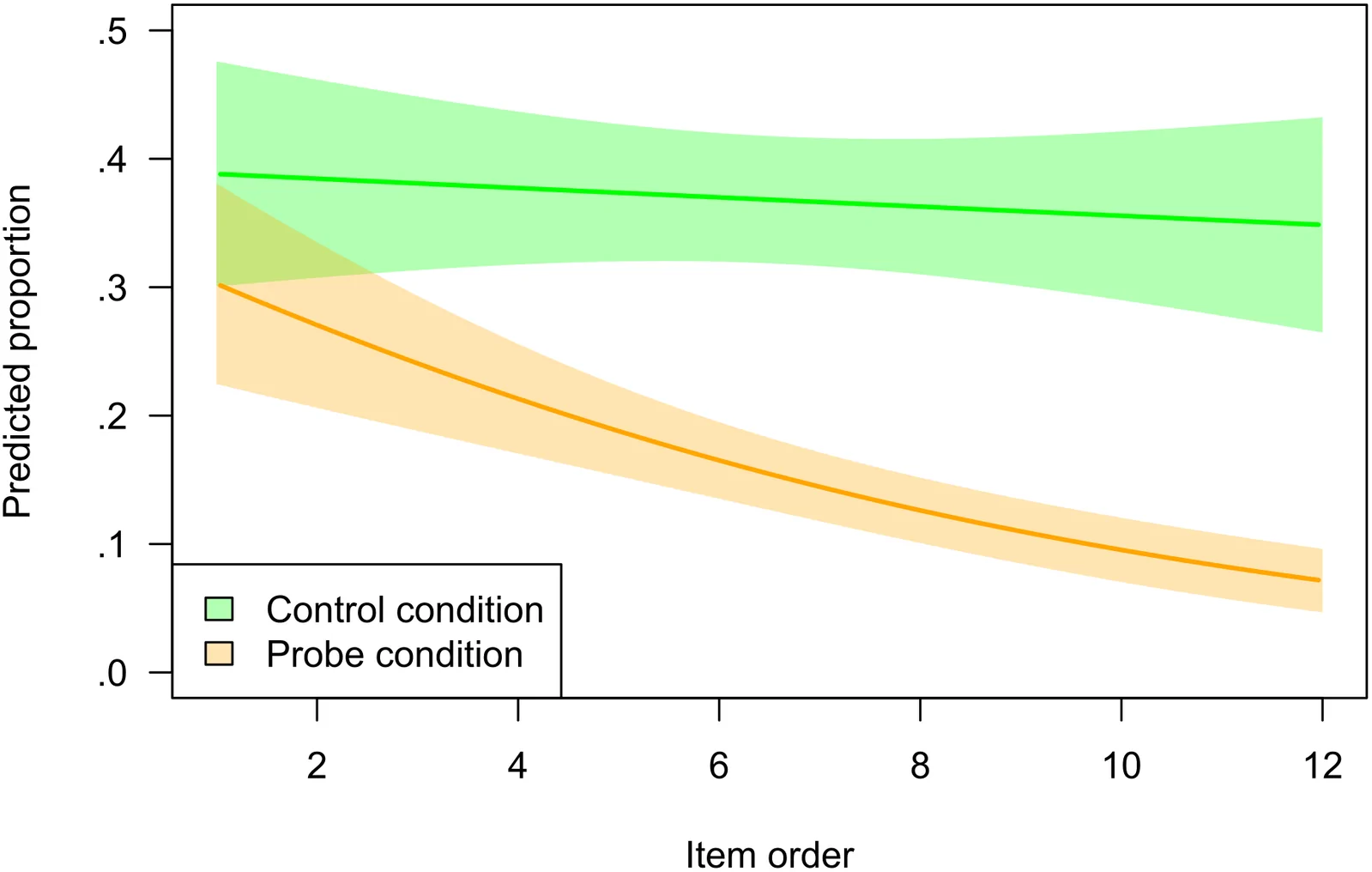
Estimated proportion giving the psychologically concerning response by the item order and condition. The shaded areas show ± one standard error. These are made just using the fixed effects.

### 3.2. Response times

The total time to complete the survey within Qualtrics was recorded as well as the time spent on each binary item of the GHQ-12. The total time is a proxy variable for the cost of the survey. The time taken with online surveys is usually positively skewed. Here the total time had a skewness of 5.33. With positively skewed data analysts often work with the geometric mean rather than the arithmetic mean. However, this should depend on the research question [22]. Here the question is framed about the total cost and therefore the arithmetic mean will be used. The mean time for the control group was 2.96 minutes (*sd* = 2.11) and for the probe group was 7.21 minutes (*sd* = 6.53). This means, on average, the control group were paid more than twice as much per hour than the probe group. The data are skewed and heteroskedastic so a bootstrap *t*-test was used [23] and confirmed the control group had a lower mean. The 95% confidence interval was (−4.84, −3.65) fewer minutes in the control condition, with an associated *p < .*001.

The individual binary item times are a way of estimating the number of mental tasks performed [24]. In the preregistration these analyses were described as exploratory and the predicted direction was not stated. Here the interest is whether there was a multiplicative shift in time duration, so the geometric means and geometric standard deviations are reported. The values in seconds per item for the two conditions and two responses were:

**Table:** 

Condition	Response	*M*_*geo*_	*SD*_*geo*_
Control	Not concerning	4.46	1.91
Control	Concerning	4.33	1.84
Probe	Not concerning	4.55	1.91
Probe	Concerning	5.55	1.94

The largest shift, about half a standard deviation above the other three cells, was for those in the probe condition who gave the psychologically concerning response being slower. A series of multilevel models were estimated predicting the natural logarithm of the item response time, as this is appropriate when examining differences among geometric means. The intercept was allowed to vary randomly for both people and items. The fixed predictors were experimental condition and whether the individual gave the psychologically concerning response. Adding the experimental condition improved the fit of the model compared with the model with just the intercept: ∆*χ*^2^(1) = 11.51*, p* = .001. Adding whether the person gave the psychologically concerning response did not significantly improve the fit compared with the model with just the intercept: ∆*χ*^2^(1) = 0.95*, p* = .329. Adding the interaction to the model with both main effects improved the fit of the model: ∆*χ*^2^(1) = 4.99*, p* = .025. These *p*-values are the unadjusted and given the more exploratory nature of these comparisons caution is urged interpreting this effect.

Further explorations were done including looking at whether item response times varied during the scale administration. Beginning with the model with the interaction (and their main effects) of experimental condition and whether the psychological concerning response was given, the item order (1–12) was added to the model and improved the fit: ∆*χ*^2^(1) = 9.04*, p* = .003. Adding the interaction between order and either the experimental condition (∆*χ*^2^(1) = 27.43*, p < .*001) or their response (∆*χ*^2^(1) = 27.43*, p < .*001) improved the fit. The three-way interaction was also significant: ∆*χ*^2^(1) = 8.56*, p* = .003. Fig 2 shows the predicted geometric means for the conditions by the item order. The largest effect is the overall decrease in response time over time, and this decrease is largest for those in the probe condition who provided the psychological concerning response. Following Spiegelhalter [25], because these are exploratory analyses they will not be discussed further.

**Fig 2:**
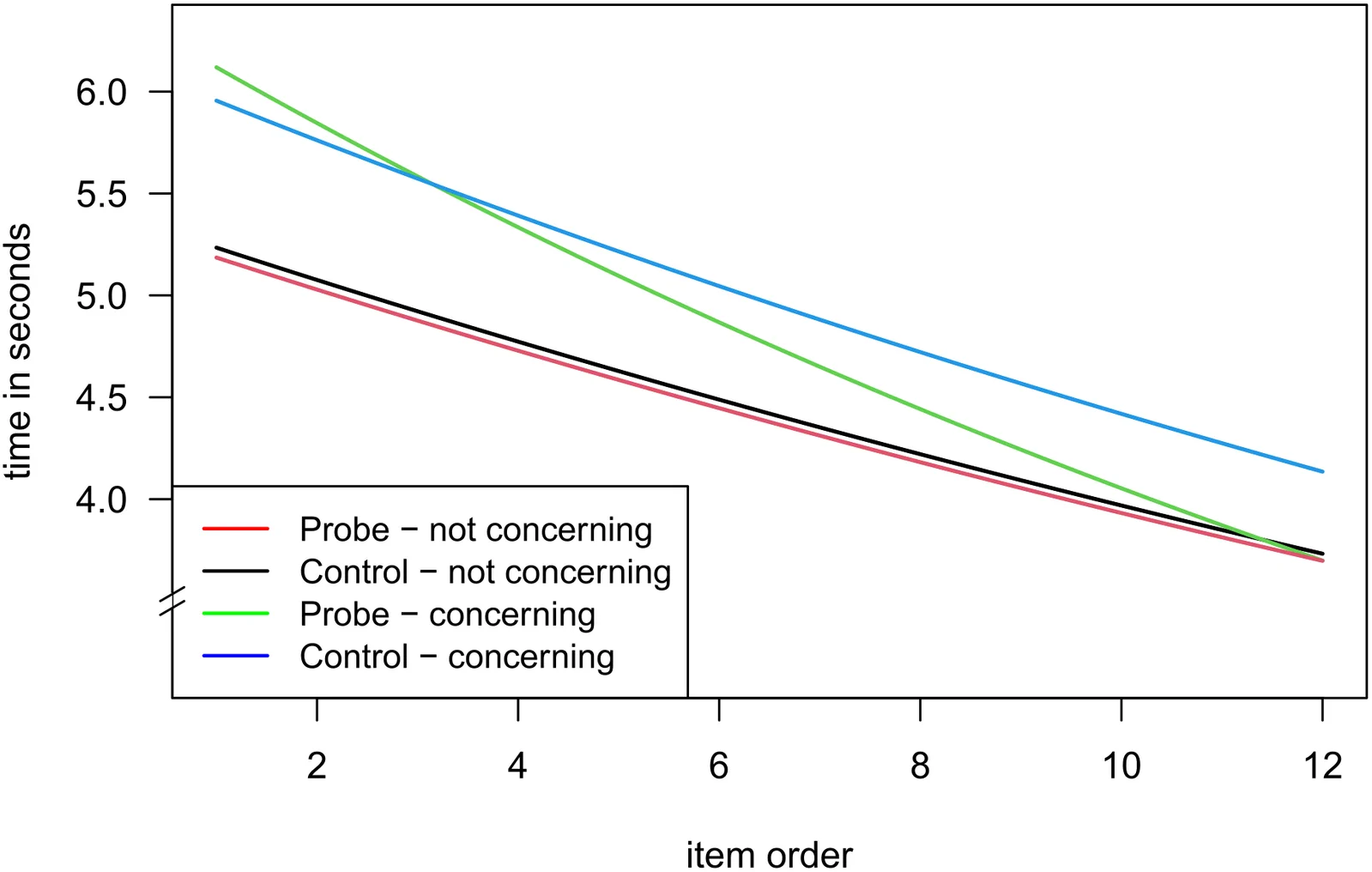
Estimated item time by the item order, experimental condition, and whether the participants provided the psychologically concerning response or not. These are made just using the fixed effects.

## 4. Discussion

Forced choice responding limits the information a person can give and interviewers will often want more information about some responses. Survey designers can include follow-up probes for responses where it is felt further information would be useful. The issue investigated here is whether this could affect the responses for other items. An online sample filled out a binary response version of the General Health Questionnaire (GHQ-12). A random half had a follow-up probe after each item where they gave the psychologically concerning response. This led to those in the probe condition giving fewer of the psychologically concerning responses than those in the control condition (prediction #1). This is consistent with people responding in a manner to avoid having to answer more questions. Answering the follow-up probes took time and those in the control condition got paid, per hour, about twice the amount of those in the probe condition. The differences in responding to the binary items increased throughout the scale administration (prediction #2). By the final few items (see Table 1 and Fig 1) people in the control condition were about twice as likely to report the psychologically concerning response.

Simon’s (1956) work on people and organizations not using optimal strategies was ground-breaking in behavioral economics. Krosnick [9] showed that participants in surveys also do not use the optimal strategies to answer the survey items correctly. Here, it can be argued the expected “correct” response proportions for the binary GHQ-12 items for the probe condition should similar to the proportions in the control condition as random assignment was used to allocate people into conditions. One possibility is that not all of those in the probe condition were using a strategy to optimize performance on the task of answering these items correctly. For some it may be that their strategy was more aligned with finishing the task rapidly while still answering in a manner that appears conscientious, but with less concern about being correct. In this sense, they were optimizing but for a different goal. Answering probes that require free response take time to respond to and survey designers should consider this cost in relation to the amount of information gained [26]. In addition, Luebker et al. [27] and Hadler [28] found including probes increased the number of people who quit the survey (i.e., break offs).

There are other possibilities. One possibility is that having thought about a psychological concerning episode in response to a previous item that their retrieval of psychological concerning episode to a new prompt could be hampered [29]. Another possibility is that taking part in previous probes affects the threshold the respondent uses to believe they should give the psychological concerning responses. Research on behavioral frequency questions shows that the threshold can be changed by aspects of the question (e.g., the response alternatives) for vaguely defined events like being annoyed [30]. All the events described in the GHQ-12 (e.g., “under strain”, “feeling unhappy”) are vague descriptions. Another possibility is that taking part in the probes makes respondents think more about the items rather than just quickly providing one of the two responses. The data reported here do not directly address which of these, or others, accounts for the results.

This experiment used a popular method for conducting psychological research, online crowd sourcing platforms, that many report can produce high quality data [e.g., 31–34]. Prolific was chosen as this platform has been shown to perform well compared with these other platforms [35]. This research shows that caution should be urged if the study has an easily recognized way to make the task take less time while still performing in a manner that would appear satisfactory. It would be worth exploring if the current findings generalize to other administrative modes [see [36], for discussion of differences in survey effects by administrative mode]. Satisficing occurs in other administrative modes [9], but the size of effects may differ. It may be that using fewer probes or making them not dependent on responses (e.g., having probes also for the non-psychologically concerning response if using this with the GHQ) could lessen effects. More research is necessary also to understand if the effects generalize to more well-defined events, like whether you ate an apple that day on a food survey or have been hospitalized, and to other forms of the GHQ.

Responses to probes can provide valuable information beyond what can be gathered from a tick mark next to a response alternative. This study does not mean that probe questions should always be avoided. In many clinical settings the interviewer uses instruments like the GHQ to structure a conversation, and this seems sensible to continue. Many short form scales are designed with this use in mind [e.g., 2]. The issue is for researchers who should be weary comparing results from the same scale if one administration uses probes and another does not, and also be aware that as the survey goes on people’s response strategies may change.
