# Supplementary material for: Asking follow-up probes for selected responses in survey scales can affect responses to these items
Source: PLoS One. 2026 Oct 1;21(10):e0359515. doi: 10.1371/journal.pone.0359515 (PMC13630244; doi:10.1371/journal.pone.0359515)
Supplement: Supplementary file 1 [file pone.0359515.s001.docx]

**Peer Review Report**

**Manuscript Title:** Asking follow-up probes for selected items in survey scales can affect responses to these items

**1. Summary of the Work**

The manuscript addresses an important methodological issue in survey design: whether adding mandatory open-ended follow-up probes to specific scale responses induces response bias (e.g., satisficing to minimize participant burden). Using a randomized experiment (n = 1,048) comparing a standard binary 12-item GHQ-12 against a version requiring a ≥ 50-character free-text explanation for "psychologically concerning" answers, the author demonstrates that mandatory probes significantly reduce the endorsement of probed responses (d ≈ 0.51). Crucially, this effect magnifies over the course of the questionnaire, illustrating a clear learning effect where participants adapt choices to avoid additional work.

**2. General Evaluation**

- **Relevance & Impact:** Highly relevant for epidemiologists, survey methodologists, and clinical researchers who frequently embed qualitative probes or logic skips into quantitative screening tools.
- **Methodological Rigor:** Strong experimental design (between-subjects randomized trial) with appropriate sample size powering (n = 1,048) and clear operationalization of variables.
- **Open Science:** Excellent adherence to open science practices, including IRB exemption documentation, pre-registration, and public hosting of de-identified data.

**3. Key Strengths**

- **Clear Mechanism & Interaction Effect:** The item-order interaction analysis (β = -0.14, p < .001) provides compelling evidence of participant task-shortening strategies over time, rather than a fixed baseline difference.
- **Practical Implications:** Highlighting the time cost (mean 7.21 min vs. 2.96 min) directly quantifies participant burden, making a strong case for survey optimization.

**4. Major Recommendations & Comments for the Author**

1. **Construct Validity vs. Measurement Error:**
   - *Comment:* The reduction in "concerning" responses could stem from two distinct mechanisms: (a) false negatives introduced in the probe condition due to satisficing, or (b) false positives reduced in the control condition because forced character entry deters non-serious or careless responses.
   - *Recommendation:* Clarify whether the study design allows for differentiating between these two possibilities, or discuss this duality explicitly in the limitations/discussion section.
2. **Generalizability Across Response Formats:**
   - *Comment:* The study utilizes a binary adaptation of the GHQ-12 with a strict 50-character minimum threshold.
   - *Recommendation:* Expand the discussion on how these findings extend to continuous/Likert scales or settings with shorter/optional character thresholds, where the burden threshold may differ.
3. **Implications for Clinical Screening:**
   - *Comment:* In public health and clinical research, conditional probes are often deployed for risk monitoring (e.g., adverse event reporting or active symptom surveillance).
   - *Recommendation:* Include concrete design recommendations for applied researchers (e.g., placing qualitative probes at the very end of a survey battery rather than inline) to mitigate the observed order interaction.

**5. Recommendation**

**Accept with Minor Revisions.** The study is well-designed, statistically sound, and directly addresses a critical gap in survey methodology. Addressing the nuances between false negatives and false positives, along with expanding practical guidance for survey designers, will further strengthen the manuscript.
