# Supplementary material for: Asking follow-up probes for selected responses in survey scales can affect responses to these items
Source: PLoS One. 2026 Oct 1;21(10):e0359515. doi: 10.1371/journal.pone.0359515 (PMC13630244; doi:10.1371/journal.pone.0359515)
Supplement: Supplementary file 2 [file pone.0359515.s002.docx]

Letter on Revisions

Thank you for the opportunity to re-submit the manuscript “Asking follow-up probes for selected responses in survey scales can affect responses to these items” to PLOS ONE (PONE-D-26-31716), and thanks to you and the reviewers from helpful comments. I will go through the comments and say where it is addressed in the revised manuscript.

1. Provide additional details regarding participant consent.

Section 2.1 on p. 6 has been added and provides information about the information given to potential participants and how they gave consent.

2. Data availability

Section 2.9 on pp. 9-10 provides the location of the data on github. The data file is time locked.

3. Ethics committee.

This information is provided in Section 2.9 on p. 9.

Reviewers.

4. The study relies on Prolific.

The reason for using Prolific is because it is becoming widely used and reviews with other online sampling companies it comes out favorably. This is discussed on pages 4 and 18. The reference on it comparing favorably is Douglas et al. (2023, PLOS ONE). It is discussed how this and similar platforms may be more likely to lead to optimizing than face-to-face surveys, or other administrative modes, but as discussed on p. 17, Krosnick (1991, ACP) has shown this occurs in other modes. On pages 18 and 19 there is more discussion that it would be valuable to see how these effects vary across modes and content areas.

5. Other explanations.

Good point. That should have been discussed and now is on page 18. One possibility is that forcing the respondent to answer a probe changes the threshold for claiming that the event is concerning. The Gaskell et al. (1994, POQ) reference is added to give an example where aspects of a survey can change the meaning of vaguely defined words (e.g., annoying). The reviewer states the effect may increase or decrease accuracy, and that is true. On p. 17 the following is said: "Here, it can be argued the expected “correct” response proportions for the binary GHQ-12 items for the probe condition should similar to the proportions in the control condition as random assignment was used to allocate people into conditions." Correct is in quotes as the items are all vague phrases (as is the norm in psychological self-report scales) and it states that it can be argued control condition is more correct rather than the control condition values are more correct. In the discussion it is stated the difficulty is comparing results when using probes from when not using probes, so not stating either is more accurate.

6. Only the binary GHQ-12 was used.

It is discussed on p. 18 the value of looking at other scales and situations where probes are sometimes used. One example mentioned is of dietary surveys where people are often asked if they had a food and then asked about proportion size and/or other details.

7. Clinical implications.

This was good that the reviewer pointed this out because it is good to stress. One use of some short forms is where the clinician asks questions from the short form to start a conversation. The example cited is the brief form of a birth-related PTSD scale that was designed in part with this purpose (Ayers et al, 2025, BMJ). It is discussed on p. 19 that continuing the conversation with probes should be continued. In this situation, one-on-one in-person discussion, is different from use of scales in research context.

In addition, a few grammatical errors were correct. The title was also changed (items to responses) to more accurately reflect the study. I believe this is a much-improved manuscript and all the points have been addressed.

Yours truly,

Daniel Wright
