# Supplementary material for: Asking follow-up probes for selected responses in survey scales can affect responses to these items
Source: PLoS One. 2026 Oct 1;21(10):e0359515. doi: 10.1371/journal.pone.0359515 (PMC13630244; doi:10.1371/journal.pone.0359515)
Supplement: Peer review history

# Peer review: Decision Letter 0

**DOI:** 10.1371/journal.pone.0359515.r001

**Authors:** Ali Cetin

11 Aug 2026

Dear Dr. Wright,

Thank you for submitting your manuscript to PLOS ONE. After careful consideration, we feel that it has merit but does not fully meet PLOS ONE’s publication criteria as it currently stands. Therefore, we invite you to submit a revised version of the manuscript that addresses the points raised during the review process.

Please submit your revised manuscript by Sep 10 2026 11:59PM. If you will need more time than this to complete your revisions, please reply to this message or contact the journal office at plosone@plos.org. When you're ready to submit your revision, log on to https://www.editorialmanager.com/pone/ and select the 'Submissions Needing Revision' folder to locate your manuscript file.

- A letter that responds to each point raised by the academic editor and reviewer(s). You should upload this letter as a separate file labeled 'Response to Reviewers'.
- A marked-up copy of your manuscript that highlights changes made to the original version. You should upload this as a separate file labeled 'Revised Manuscript with Track Changes'.
- An unmarked version of your revised paper without tracked changes. You should upload this as a separate file labeled 'Manuscript'.

If you would like to make changes to your financial disclosure, please include your updated statement in your cover letter. Guidelines for resubmitting your figure files are available below the reviewer comments at the end of this letter.

If applicable, we recommend that you deposit your laboratory protocols in protocols.io to enhance the reproducibility of your results. Protocols.io assigns your protocol its own identifier (DOI) so that it can be cited independently in the future. For instructions see: https://journals.plos.org/plosone/s/submission-guidelines#loc-laboratory-protocols. Additionally, PLOS ONE offers an option for publishing peer-reviewed Lab Protocol articles, which describe protocols hosted on protocols.io. Read more information on sharing protocols at https://plos.org/protocols?utm_medium=editorial-email&utm_source=authorletters&utm_campaign=protocols.

As the corresponding author, your ORCID iD is verified in the submission system and will appear in the published article. PLOS supports the use of ORCID, and we encourage all coauthors to register for an ORCID iD and use it as well. Please encourage your coauthors to verify their ORCID iD within the submission system before final acceptance, as unverified ORCID iDs will not appear in the published article. *Only*  the individual author can complete the verification step; PLOS staff *cannot*  verify ORCID iDs on behalf of authors.

We look forward to receiving your revised manuscript.

Kind regards,

Ali Cetin

Academic Editor

PLOS One

**Journal Requirements:**

When submitting your revision, we need you to address these additional requirements.

1. Please ensure that your manuscript meets PLOS ONE's style requirements, including those for file naming. The PLOS ONE style templates can be found at

https://journals.plos.org/plosone/s/file?id=wjVg/PLOSOne_formatting_sample_main_body.pdf and

https://journals.plos.org/plosone/s/file?id=ba62/PLOSOne_formatting_sample_title_authors_affiliations.pdf

2. Please provide additional details regarding participant consent. In the ethics statement in the Methods and online submission information, please ensure that you have specified (1) whether consent was informed and (2) what type you obtained (for instance, written or verbal, and if verbal, how it was documented and witnessed). If your study included minors, state whether you obtained consent from parents or guardians. If the need for consent was waived by the ethics committee, please include this information.

3. If you are reporting a retrospective study of medical records or archived samples, please ensure that you have discussed whether all data were fully anonymized before you accessed them and/or whether the IRB or ethics committee waived the requirement for informed consent. If patients provided informed written consent to have data from their medical records used in research, please include this information.

4. Please note that your Data Availability Statement is currently missing the repository name. If your manuscript is accepted for publication, you will be asked to provide these details on a very short timeline. We therefore suggest that you provide this information now, though we will not hold up the peer review process if you are unable.

5. Please include your full ethics statement in the ‘Methods’ section of your manuscript file. In your statement, please include the full name of the IRB or ethics committee who approved or waived your study, as well as whether or not you obtained informed written or verbal consent. If consent was waived for your study, please include this information in your statement as well.

6. Please include captions for your Supporting Information files at the end of your manuscript, and update any in-text citations to match accordingly. Please see our Supporting Information guidelines for more information: http://journals.plos.org/plosone/s/supporting-information.

7. If the reviewer comments include a recommendation to cite specific previously published works, please review and evaluate these publications to determine whether they are relevant and should be cited. There is no requirement to cite these works unless the editor has indicated otherwise.

8. Please review your reference list to ensure that it is complete and correct. If you have cited papers that have been retracted, please include the rationale for doing so in the manuscript text, or remove these references and replace them with relevant current references. Any changes to the reference list should be mentioned in the rebuttal letter that accompanies your revised manuscript. If you need to cite a retracted article, indicate the article’s retracted status in the References list and also include a citation and full reference for the retraction notice.

[Note: HTML markup is below. Please do not edit.]

Reviewers' comments:

Reviewer's Responses to Questions

**Comments to the Author**

1. Is the manuscript technically sound, and do the data support the conclusions?

Reviewer #1: Yes

Reviewer #2: Yes

**********

2. Has the statistical analysis been performed appropriately and rigorously?

Reviewer #1: Yes

Reviewer #2: Yes

**********

3. Have the authors made all data underlying the findings in their manuscript fully available?

The PLOS Data policy

Reviewer #1: Yes

Reviewer #2: Yes

**********

4. Is the manuscript presented in an intelligible fashion and written in standard English?

Reviewer #1: Yes

Reviewer #2: Yes

**********

Reviewer #1: Dear Author,

Please address the following queries satisfactorily-

1.The study relies exclusively on participants recruited through Prolific, limiting the generalizability of the findings to broader populations, particularly individuals with lower digital literacy or those assessed in face-to-face clinical settings.

2.The explanation that participants altered responses primarily to avoid additional effort remains inferential because participant motivation was not directly measured. Alternative explanations, such as increased self-reflection induced by follow-up probes, cannot be ruled out.

3.Only the binary version of the GHQ-12 was examined, making it uncertain whether similar effects would occur with Likert-type scales or other psychological instruments.

Reviewer #2: The study is well-designed, statistically sound, and directly addresses a critical gap in survey methodology. Addressing the nuances between false negatives and false positives, along with expanding practical guidance for survey designers, will further strengthen the manuscript.

**********

what does this mean?). If published, this will include your full peer review and any attached files.

If you choose “no”, your identity will remain anonymous but your review may still be made public.

**Do you want your identity to be public for this peer review?**  For information about this choice, including consent withdrawal, please see our Privacy Policy

Reviewer #1: No

Reviewer #2: **Yes:**  Juliana Aggrey

**********

[NOTE: If reviewer comments were submitted as an attachment file, they will be attached to this email and accessible via the submission site. Please log into your account, locate the manuscript record, and check for the action link "View Attachments". If this link does not appear, there are no attachment files.]

To ensure your figures meet our technical requirements, please review our figure guidelines: https://journals.plos.org/plosone/s/figures

You may also use PLOS’s free figure tool, NAAS, to help you prepare publication quality figures: https://journals.plos.org/plosone/s/figures#loc-tools-for-figure-preparation.

NAAS will assess whether your figures meet our technical requirements by comparing each figure against our figure specifications.

Attachment Submitted filename: PONE-D-26-31716.docx

# Author comment: Author response to Decision Letter 1

**DOI:** 10.1371/journal.pone.0359515.r002

27 Aug 2026

Dear Dr Ali Cetin,

Thank you for the opportunity to re-submit the manuscript “Asking follow-up probes for selected responses in survey scales can affect responses to these items” to PLOS ONE (PONE-D-26-31716), and thanks to you and the reviewers from helpful comments. I will go through the comments and say where it is addressed in the revised manuscript.

1. Provide additional details regarding participant consent.

Section 2.1 on p. 6 has been added and provides information about the information given to potential participants and how they gave consent.

2. Data availability

Section 2.9 on pp. 9-10 provides the location of the data on github. The data file is time locked.

3. Ethics committee.

This information is provided in Section 2.9 on p. 9.

Reviewers.

4. The study relies on Prolific.

The reason for using Prolific is because it is becoming widely used and reviews with other online sampling companies it comes out favorably. This is discussed on pages 4 and 18. The reference on it comparing favorably is Douglas et al. (2023, PLOS ONE). It is discussed how this and similar platforms may be more likely to lead to optimizing than face-to-face surveys, or other administrative modes, but as discussed on p. 17, Krosnick (1991, ACP) has shown this occurs in other modes. On pages 18 and 19 there is more discussion that it would be valuable to see how these effects vary across modes and content areas.

5. Other explanations.

Good point. That should have been discussed and now is on page 18. One possibility is that forcing the respondent to answer a probe changes the threshold for claiming that the event is concerning. The Gaskell et al. (1994, POQ) reference is added to give an example where aspects of a survey can change the meaning of vaguely defined words (e.g., annoying). The reviewer states the effect may increase or decrease accuracy, and that is true. On p. 17 the following is said: "Here, it can be argued the expected “correct” response proportions for the binary GHQ-12 items for the probe condition should similar to the proportions in the control condition as random assignment was used to allocate people into conditions." Correct is in quotes as the items are all vague phrases (as is the norm in psychological self-report scales) and it states that it can be argued control condition is more correct rather than the control condition values are more correct. In the discussion it is stated the difficulty is comparing results when using probes from when not using probes, so not stating either is more accurate.

6. Only the binary GHQ-12 was used.

It is discussed on p. 18 the value of looking at other scales and situations where probes are sometimes used. One example mentioned is of dietary surveys where people are often asked if they had a food and then asked about proportion size and/or other details.

7. Clinical implications.

This was good that the reviewer pointed this out because it is good to stress. One use of some short forms is where the clinician asks questions from the short form to start a conversation. The example cited is the brief form of a birth-related PTSD scale that was designed in part with this purpose (Ayers et al, 2025, BMJ). It is discussed on p. 19 that continuing the conversation with probes should be continued. In this situation, one-on-one in-person discussion, is different from use of scales in research context.

In addition, a few grammatical errors were correct. The title was also changed (items to responses) to more accurately reflect the study. I believe this is a much-improved manuscript and all the points have been addressed.

Yours truly,

Daniel Wright

Attachment Submitted filename: revisionletter.docx

# Editor report: Decision Letter 1

**DOI:** 10.1371/journal.pone.0359515.r003

**Authors:** Ali Cetin

15 Sep 2026

Asking follow-up probes for selected responses in survey scales can affect responses to these items

PONE-D-26-31716R1

Dear Dr. Wright,

We’re pleased to inform you that your manuscript has been judged scientifically suitable for publication and will be formally accepted for publication once it meets all outstanding technical requirements.

Within one week, you’ll receive an e-mail detailing the required amendments. When these have been addressed, you’ll receive a formal acceptance letter and your manuscript will be scheduled for publication.

An invoice will be generated when your article is formally accepted. Please note, if your institution has a publishing partnership with PLOS and your article meets the relevant criteria, all or part of your publication costs will be covered. Please make sure your user information is up-to-date by logging into Editorial Manager at Editorial Manager® and clicking the ‘Update My Information' link at the top of the page. For questions related to billing, please contact billing support.

If your institution or institutions have a press office, please notify them about your upcoming paper to help maximize its impact. If they’ll be preparing press materials, please inform our press team as soon as possible -- no later than 48 hours after receiving the formal acceptance. Your manuscript will remain under strict press embargo until 2 pm Eastern Time on the date of publication. For more information, please contact onepress@plos.org.

Kind regards,

Ali Cetin

Academic Editor

PLOS One

Additional Editor Comments (optional):

Reviewers' comments:

# Editor report: Acceptance letter

**DOI:** 10.1371/journal.pone.0359515.r004

**Authors:** Ali Cetin

PONE-D-26-31716R1

PLOS One

Dear Dr. Wright,

I'm pleased to inform you that your manuscript has been deemed suitable for publication in PLOS One. Congratulations! Your manuscript is now being handed over to our production team.

At this stage, our production department will prepare your paper for publication. This includes ensuring the following:

* All references, tables, and figures are properly cited

* All relevant supporting information is included in the manuscript submission,

* There are no issues that prevent the paper from being properly typeset

You will receive further instructions from the production team, including instructions on how to review your proof when it is ready. Please keep in mind that we are working through a large volume of accepted articles, so please give us a few days to review your paper and let you know the next and final steps.

Lastly, if your institution or institutions have a press office, please let them know about your upcoming paper now to help maximize its impact. If they'll be preparing press materials, please inform our press team within the next 48 hours. Your manuscript will remain under strict press embargo until 2 pm Eastern Time on the date of publication. For more information, please contact onepress@plos.org.

You will receive an invoice from PLOS for your publication fee after your manuscript has reached the completed accept phase. If you receive an email requesting payment before acceptance or for any other service, this may be a phishing scheme. Learn how to identify phishing emails and protect your accounts at https://explore.plos.org/phishing.

If we can help with anything else, please email us at customercare@plos.org.

Thank you for submitting your work to PLOS One and supporting open access.

Kind regards,

PLOS One Editorial Office Staff

on behalf of

Professor Ali Cetin

Academic Editor

PLOS One
